# Work- and stress-related musculoskeletal and sleep disorders among health professionals: a cross-sectional study in a hospital setting in Switzerland

**DOI:** 10.1186/s12891-020-03327-w

**Published:** 2020-05-21

**Authors:** Oliver Hämmig

**Affiliations:** Epidemiology, Biostatistics and Prevention Institute, University of Zurich, Hirschengraben 84, 8001 Zurich, Switzerland

**Keywords:** Musculoskeletal disorders, Sleep disorders, Physical workload, Psychological stress, Hospital workers, Nurses, Switzerland

## Abstract

**Background:**

Musculoskeletal and sleep disorders have been reported to be very common among health care and hospital workers and particularly nurses. They are assumed or found to be a result of psychological stress and/or physical strain or pain. However, no other study so far – at least in a hospital setting and for Switzerland – has considered and investigated musculoskeletal as well as sleep disorders in consequence of or rather in association with both physical workload and psychological stress.

**Methods:**

Cross-sectional survey data of 1232 health professionals were used and analysed. Data were collected in 2015/16 among the health care workforces of three public hospitals and two rehabilitation clinics in the German-speaking part of Switzerland. Musculoskeletal and sleep disorders were assessed by three items taken from the Swiss Health Survey, a 2-item measure of accumulated low back, back, neck and shoulder pain and a single-item measure of problems in getting to sleep or sleeping through. Stratified and adjusted bivariate logistic and multivariate linear regression analyses were performed to calculate measures of association (adjusted odds ratios, z-standardized beta coefficients), to control for potential confounders, and to compare different health professions (nurses, physicians, therapists, other).

**Results:**

Almost every fourth of the studied health professionals reported severe or even very severe musculoskeletal disorders (MSDs) and nearly every seventh severe sleep disorders (SDs). These prevalence rates were significantly or at least slightly higher among nurses than among physicians and other health care workers. General stress, work stress, physical effort at work, and particularly a painful or tiring posture at work were found to be clear and strong risk factors for MSDs, whereas only general and work-related stress were found to be significantly associated with SDs. There was no or only weak association between MSDs and SDs.

**Conclusions:**

This study found MSDs to be largely a result of physical workload or rather poor posture at work and only secondarily a consequence of (general) stress, whereas SDs were revealed to be primarily a consequence of stress on and particularly off the job. Preventive strategies therefore have to differentiate and combine measures for the reduction of both psychological stress and physical strain.

## Background

It is well-known in occupational medicine that musculoskeletal injuries and disorders (MSDs) are strongly work-related and as such one of the most prevalent occupational diseases in modern societies and working populations. According to the European Foundation for the Improvement of Living and Working Conditions, MSDs are the main occupational disease suffered by European workers and account for more than 50% of serious work-related diseases [1]. Furthermore, it has been widely reported that MSDs are particularly prevalent and among the most common health complaints in health care workers and especially in hospital staff and among nurses and physical therapists [2–12].

Research in occupational medicine has identified a number of physical and psychosocial risk factors for the development of work-related MSDs [13–23]. Previous studies have shown that MSD are directly caused by physically demanding work and strenuous working conditions, such as lifting or carrying heavy loads, tiring positions, awkward posture, or repetitive movements [13–15]. Moreover, MSDs were also found to be related and associated with psychologically stressful work, i.e. with psychosocial work factors and work-related stressors such as time pressure, low job control, little social or supervisor support, effort-reward imbalance, or work-life conflict [16–23].

In spite of such extensive research literature, only very few studies have investigated MSDs depending on both physical workload *and* psychological stress and – if at all – mostly looked at specific pain (e.g. neck or shoulder pain, upper limb pain, low back pain) and/or individual occupational groups (predominantly nurses in direct patient care) and none for Switzerland. The same applies to sleep problems or disorders, which were found not only to be very common among hospital workers and particularly nurses [24, 25], to have similar (psychosocial) risk factors as MSDs [26–28] and to be likewise associated with (work) stress [27, 28], but also to be interrelated with (musculoskeletal) pain [29–31]. And although stress and pain in general and work stress and musculoskeletal pain in particular have been identified as predictors or correlates of poor sleep, only few studies have investigated stress-related and painful MSDs jointly with sleep disorders (SDs).

In light of all the studied associations and mentioned limitations of previous research in this field, this study, which is situated in a health care setting in Switzerland, investigates both musculoskeletal *and* – subsequently – sleep disorders as a possible result of or rather in association with both physical (work) load *and* psychological (work) stress. The study thereby takes into account not only nurses but also physicians, therapists, and other health professionals working in public hospitals and rehab clinics. In addition, the study does not focus on single musculoskeletal pains but on multiple and combined neck, shoulder, arm, back, and low back pain. Furthermore, SDs are considered as related with MSDs or rather as a consequence of (musculoskeletal) pain, as has been repeatedly reported previously [29, 31].

**Fig. 1:**
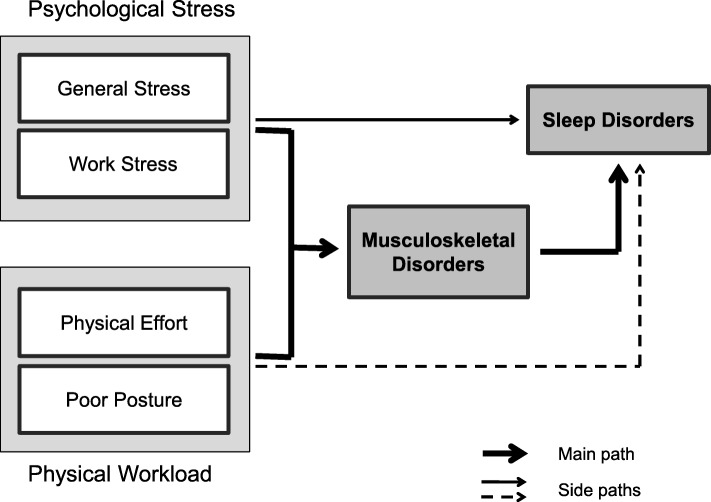
Theoretical path model explaining musculoskeletal and sleep disorders

The path model (see Fig. 1) illustrates the assumed causal paths und underlying hypotheses of the present study: Psychological stress and physical workload are expected to strongly determine and equally cause musculoskeletal pain or disorders, which in turn are hypothesized to produce sleep problems. In other words, MSDs are directly and strongly caused by physical workload and psychological (work) stress, whereas SDs are expected to be only or mainly indirectly caused by physical workload and psychological (work) stress and to result directly from MSDs.

This study addressed the following research questions:

- Are physical workloads and/or psychological stresses and therefore musculoskeletal and sleep disorders more common among nurses than among other health professionals as expected?
- Can a (similarly) strong association be observed between both psychological stress and physical workload on the one hand and MSDs on the other? Is there at the same time a much less strong relation between these conditions and SDs as hypothesized?
- Do SDs go along with MSDs as assumed? And if so, are SDs substantially more strongly associated with MSDs than with psychological stress or physical workload?

## Methods

### Data and study sample

Secondary cross-sectional survey data were used for this study, i.e. data that already existed and were collected between summer 2015 and spring 2016. The survey was conducted in a health care setting, or more precisely, among the workforces of initially four public hospitals and two rehabilitation clinics in German-speaking Switzerland. The original purpose of the survey was to broadly and exploratively study the working conditions, workloads and health of healthcare workers and particularly health professionals. The only inclusion criterion for the survey participation was being employed by one of the six selected healthcare institutions at the period of the data collection. In total, 1840 hospital employees participated voluntarily and anonymously in the full sample postal survey and filled in a written questionnaire containing 100 questions on “Work and Health in the Hospital”. The anonymous data collection did not allow participants to be identified. The return rate overall was 41% and ranged from 36 to 49% for the participating hospitals and rehab clinics, which included a university hospital, a cantonal hospital, and two regional or district hospitals.

Due to the accidental absence of a single-item and 5-point scaled measure of general psychological stress in the written questionnaire for one hospital, the present study was limited to five of the six originally participating hospitals and rehab clinics, excluding one district hospital with 273 employees who participated in the survey. An additional 335 hospital employees and members from other than health professions were excluded from the study. The study population was therefore restricted to a total of 1232 health professionals, including 718 nurses and midwives (58.3% of the study sample), 222 physicians (18.0%), 137 therapists (11.1%), and 155 other health professionals (12.6%) such as medical technical staff, scientific staff, etc. Being a health professional and having answered to the general stress question were the two selection criteria for getting included in the study population.

Due to small numbers of cases and for anonymity reasons, individual health professions were summarized in larger professional categories, except for nurses and physicians, the two numerically largest and most homogeneous single health professions in the study population by far. Such broad categorization or rough classification into four sufficiently large occupational groups was done not only for the data analysis and the present study but largely already in the survey when response categories for the question “To which profession do you belong?” were specified. Nurses and midwives were summarized in a single category, since only 21 midwives participated in the survey. All kinds of therapists (physiotherapists, psychotherapists, occupational therapists etc.) were also merged into one professional group. And medical-technical staffs (radiologists, lab assistants, etc.) or scientific and academic staffs (psychologists, pharmacists, biologists, etc.) were summarized in the residual and heterogeneous category “other health professionals”.

More than 87% of the study population were women, with a share of approximately 95% among nurses and midwives, nearly 84% among therapists, and more than 65% among physicians. The selected study participants were mostly highly educated (76.5%), with higher vocational education, a maturity certificate, or even a university degree, and were below age 45 (65.7%).

### Measures

#### Musculoskeletal disorders

MSDs were assessed by a question and two items taken from the periodically conducted Swiss Health Survey: “Please indicate if and how pronounced you’ve had any of the following complaints in the last four weeks: a) back pain or pain in the lower back and b) neck ache or shoulder pain”. For each of these complaints, the response options were “not at all” (score 0), “a little” (1), or “severe” (2). A summary score was created by adding up the scores of the two 3-point scaled items to an overall MSD scale ranging from 0 to 4 and indicating accumulated and (very) severe MSDs with scores of 3 or 4.

#### Sleep disorders

SDs are defined as difficulties in initiating and/or maintaining sleep (sleep-onset or maintenance insomnia) and were assessed by the question “Please indicate if and how pronounced you’ve had any of the following complaints in the last four weeks: difficulties in falling or staying asleep”, with response options from 0 “not at all”, 1 “a little”, to 2 “severe”.

#### General stress

General stress, defined as universal psychological stress (as distinguished from physiological stress), was assessed by an established and validated single-item measure of psychological stress symptoms developed in the early 1970s [32]. The questionnaire provided a definition of stress (“Stress means a situation in which a person feels tense, restless, nervous and anxious and/or is unable to sleep at night because his/her mind is troubled all the time”) and then included the following question: “Do you feel this kind of stress currently – and to what extent?” The response options on a 5-point scale ranged from 0 “not at all” to 4 “very strongly”.

#### Work stress

Work stress, understood as psychological stress on the job or related to the job, was assessed by an established and widely used multiple-item measure of failed reciprocity or gratification crisis at work, better known as effort-reward imbalance (ERI). This imbalance is conceptualized as a perceived lack of reward received from or at work compared to the effort put into work [33] and was measured by a short version of the ERI questionnaire consisting of two subscales (effort, reward) and a total of 16 items [34]. The so-called ERI ratio then was calculated from the two 10-item and 6-item subscales or sum scores of “effort” (numerator) and “reward” (denominator), multiplied by a factor that corrects for the different numbers of items of the two subscales. The ERI ratio quantifies the amount of imbalance or reward frustration and stress at work. In general, the amount of imbalance or work stress increases with increasing values of the ERI ratio. In particular, a ratio of below one indicates an unproblematic imbalance for the benefit of the reward component while a ratio of more than one indicates an under-rewarded work effort or rather a stressful high effort / low reward job situation.

#### Physical workload

Physically demanding work was assessed by two separate measures concerning physical effort and poor posture at work. Physical effort at work was measured by a scale consisting of three items on the extent one’s work requires carrying or moving persons, carrying or moving heavy loads or standing (response options from 0 “never” to 4 “permanently”). A combined score has been calculated with a range of values from 0 to 12 and a Cronbach’s alpha of .66 (as a measure of internal consistency). Poor posture at work was analogously measured with a single item: “Please indicate to what extent your work includes the following: painful or tiring posture” (response options from 0 “never” to 4 “permanently”).

### Analyses

First, relative frequencies of hypothesized risk factors or exposures (general stress, work stress, physical effort at work, poor posture at work) and prevalence rates of health outcomes (MSDs, SDs) were calculated, aggregated for all health professionals, and stratified for specific health professions (nurses, physicians, therapists, other).

Second, crosstabulations and bivariate logistic regression analyses were performed in order to estimate relative frequencies and odds ratios as measures of the relative risks of physically demanding and psychologically stressful working conditions related to MSDs and SDs. These bivariate association analyses were adjusted for control variables such as sex, age, and education.

Finally, multivariate linear regression analyses were carried out to calculate z-standardized and multiple adjusted path or beta coefficients and to obtain independent and comparable effects of the assumed main work-related and stress-related predictors in explaining MSDs and SDs. In accordance with the theoretical path model (see Fig. 1) which postulates only direct effects of psychological stress and physical workload on MSDs, but also and mainly indirect effects (via MSDs) on SDs, stepwise (and stratified) linear regression analyses were performed for explaining or determining SDs with step 1 representing the main but indirect path and step 2 representing the side but direct path.

## Results

Regarding the first research question: Among nurses and midwives, the proportion of those who report high physical effort at work (27%) and regular to permanent poor work posture (27%) was substantially above the average of all health professionals and particularly high compared to physicians (1%, resp. 10%) and other health professionals (5%, resp. 17%) (see Table 1). Moderate to high work stress as measured by effort-reward imbalance was widely spread throughout the entire study population and clearly and significantly more prevalent among nurses (72%) than among physicians (65%) and all other health professionals (54–59%). On the other hand, moderate to (very) strong general stress was also very common among the considered health professions, but did not significantly differ between nurses (47%) and all other health professionals (50–52%), as shown in Table 1.

**Table 1:** Sociodemographic characteristics, prevalence rates of musculoskeletal and sleep disorders, general and job stress, and physical workload among health professionals

	Nurses (and midwives)		Physicians		Therapists		Other health professionals		All health professionals		
	*n* = 718		*n* = 222		*n* = 137		*n* = 155		*N* = 1232		
	**N**	**%**	**N**	**%**	**N**	**%**	**N**	**%**	**N**	**%**	
*Sex*											**
Female	675	94.5	145	65.3	115	83.9	136	87.7	**1071**	**87.2**	
Male	39	5.5	77	34.7	22	16.1	19	12.3	**157**	**12.8**	
*Age*											***
< 25 years	72	10.0	2	0.9	5	3.7	2	1.3	**81**	**6.6**	
25–34 years	221	30.8	72	32.7	52	38.9	55	35.5	**400**	**32.6**	
35–44 years	173	24.1	74	33.6	38	27.9	41	26.4	**326**	**26.5**	
45–54 years	164	22.9	45	20.5	26	19.1	38	24.5	**273**	**22.2**	
55+ years	87	12.1	27	12.3	15	11.0	19	12.3	**148**	**12.1**	
*Education* *(highest level achieved)*											***
Low (1–4)	27	3.8	0	–	0	–	0	–	**27**	**2.2**	
Medium (5–6)	213	30.2	3	1.4	12	8.8	30	19.6	**258**	**21.3**	
High (7–10)	346	49.0	2	0.9	28	20.4	51	33.3	**427**	**35.2**	
Very high (11–12)	120	17.0	212	97.7	97	70.8	72	47.1	**501**	**41.3**	
*Musculoskeletal disorders*											***
None (0)	117	16.5	66	30.3	36	26.3	41	26.5	**260**	**21.4**	
Minor to moderate (1–2)	398	56.3	115	52.8	74	54.0	81	52.3	**668**	**54.9**	
Severe to very severe (3–4)	192	27.2	37	17.0	27	19.7	33	21.3	**289**	**23.7**	
*Sleep disorders*											n.s.
Not at all (0)	340	47.9	109	49.3	56	40.9	66	42.6	**571**	**46.7**	
A little (1)	273	38.5	88	39.8	63	46.0	68	43.9	**492**	**40.2**	
Severe (2)	97	13.7	24	10.9	18	13.1	21	13.5	**160**	**13.1**	
*General stress*											n.s.
Not at all to minimal (0–1)	363	53.2	106	49.3	66	49.6	72	48.3	**607**	**51.5**	
Moderate (2)	229	33.6	69	32.1	44	33.1	55	36.9	**397**	**33.7**	
Strong to very strong (3–4)	90	13.2	40	18.6	23	17.3	22	14.8	**175**	**14.8**	
*Work stress*											***
Very low (ERI ratio ≤ 0.8)	43	6.5	23	11.3	19	14.7	31	21.1	**116**	**10.2**	
Low (ERI ratio > 0.8–1)	146	22.0	48	23.6	40	31.0	30	20.4	**264**	**23.1**	
Moderate (ERI ratio > 1–1.5)	379	57.2	108	53.2	56	43.4	68	46.3	**611**	**53.5**	
High (ERI ratio > 1.5)	95	14.3	24	11.8	14	10.9	18	12.2	**151**	**13.2**	
*Physical effort at work*											***
No / low (0–1)	86	12.6	111	51.2	53	39.6	83	56.8	**333**	**28.2**	
Medium (2–5)	416	60.8	104	47.9	57	42.5	56	38.4	**633**	**53.6**	
High (6–12)	182	26.6	2	0.9	24	17.9	7	4.8	**215**	**18.2**	
*Poor posture at work*											***
Never to occasionally (0–1)	519	73.3	194	89.8	112	82.4	123	82.6	**948**	**78.4**	
Regularly (2)	125	17.7	16	7.4	21	15.4	14	9.4	**176**	**14.6**	
Frequently to permanently (3–4)	64	9.0	6	2.8	3	2.2	12	8.1	**85**	**7.0**	

Pearson’s chi-square test: *p ≤ .05; **p < .01; ***p < .001; n.s. = not significant (*p* > .05)

As expected, strong accumulated MSDs were significantly more prevalent among nurses (27%) than among all other health professionals (17% up to 21%). In contrast, severe SDs or problems in falling or staying asleep were only slightly or not at all more frequent among nurses (14%) than among physicians (11%), therapists (13%), or other health professionals (14%), as indicated in Table 1.

Concerning the second and third research questions: Psychological stress turned out to be a very strong risk factor for MSDs and SDs. Whereas the relative risk of both severe MSDs and severe SDs increased remarkably up to an odds ratio of above 17 with increasing levels of general stress and work stress, associations were strongest and gradients were steepest for SDs in association with general stress and for MSDs in association with work stress (see Table 2).

**Table 2:** Associations of physically demanding and psychologically stressful work with musculoskeletal and sleep disorders among health professionals

	Severe musculoskeletal disorders (3–4)			Severe sleep disorders (2)		
	%	aOR^1)^	95% CI	%	aOR^1)^	95% CI
**Total study population**	23.7			13.1		
**General stress**						
Not at all to minimal (0–1)	13.0	1		3.8	1	
Moderate (2)	30.0	**3.01**	2.16–4.18	14.5	**4.36**	2.63–7.24
Strong to very strong (3–4)	48.5	**6.88**	4.61–10.28	41.1	**17.67**	10.47–29.82
* Number of cases*			*1140*			*1146*
**Work stress**						
Very low (ERI ratio ≤ 0.8)	6.0	1		6.1	1	
Low (ERI ratio > 0.8–1)	14.9	**2.61**	1.12–6.06	7.7	1.19	0.49–2.90
Moderate (ERI ratio > 1–1.5)	25.5	**5.20**	2.36–11.45	11.9	1.97	0.88–4.42
High (ERI ratio > 1.5)	44.6	**13.03**	5.64–30.08	32.0	**6.87**	2.96–15.94
* Number of cases*			*1106*			*1110*
**Physical effort at work**						
No / low (0–1)	17.8	1		13.3	1	
Medium (2–5)	22.8	1.42	0.99–2.02	11.3	0.97	0.64–1.47
High (6–12)	35.8	**2.51**	1.62–3.91	16.4	**1.72**	1.01–2.93
* Number of cases*			*1145*			*1150*
**Poor posture at work**						
Never to sometimes (0–1)	17.8	1		10.7	1	
Regularly (2)	40.6	**3.04**	2.13–4.33	20.0	**2.33**	1.51–3.60
Often to always (3–4)	58.3	**6.26**	3.88–10.09	22.6	**2.71**	1.52–4.82
* Number of cases*			*1171*			*1176*
**Musculoskeletal disorders**						
None (0)	–	–		8.1	1	
Minor to moderate (1–2)	–	**–**	–	8.7	1.19	0.70–2.02
Severe to very severe (3–4)	–	**–**	–	27.0	**4.65**	2.73–7.91
* Number of cases*			–			*1190*

^1)^ Odds ratios adjusted for sex, age, and education

Bold print = significant (*p* < = .05)

Associations with MSDs and SDs overall were significantly less accentuated for physical workload (physical effort at work, poor posture at work) than for psychological stress (general stress, work stress). But whereas general and work-related stress were found to be strong risk factors for both MSDs and SDs, physical workloads and particularly poor work posture were more strongly associated with MSDs than with SDs (see Table 2).

Against expectations, MSDs and SDs were not linearly correlated or gradually associated with each other, but as Table 2 shows, having strong accumulated MSDs strongly increased the risk of having equally strong SDs compared to those without any reported MSDs at all (OR = 4.7). However, the fairly strong bivariate association between MSDs and SDs (β = .21) decreased substantially in a multivariate association analysis (β = .07) and turned out to be largely mediated or rather confounded by (general) stress, as shown in Table 4.

Other findings from bivariate logistic regression analyses were fully supported by the results of multivariate linear regression analyses. When adjusting for all considered covariates, indicators of physical workload (physical effort at work, poor posture at work) better explained and more strongly predicted MSDs among health professionals compared to the stress measures (general stress, work stress) used (see Table 3). In turn, SDs among specific health professions or among health professionals in total were not or hardly determined by physical workload, work stress or musculoskeletal pain but were mainly and strongly associated with general stress (see Table 4).

**Table 3:** Explaining musculoskeletal disorders – results of multiple linear regression analyses

(***Dependent variable:***) **Musculoskeletal disorders** (two-item scale from 0 ‘none’ to 4 ‘very severe’)	Nurses (and midwives)	Physicians	Therapists and other health professionals	All health professionals
	*n* = 718	*n* = 222	*n* = 292	*N* = 1232
	*beta coeff. (β)*	*beta coeff. (β)*	*beta coeff. (β)*	*beta coeff. (β)*
(***Independent variables:***)				
**General stress** (single-item scale from 1 ‘not at all’ to 5 ‘very strong’)	.14***	.13	.22***	.15***
**Work stress** (ERI ratio from 0.3 ‘lowest’ to 3.7 ‘highest’)	.08	.23**	.02	.09**
**Physical effort at work** (three-item scale from 0 ‘lowest’ to 12 ‘highest’)	−.01	.07	−.05	−.01
**Poor posture at work** (single-item scale from 0 ‘never’ to 4 ‘permanently’)	.35***	.17*	.26***	.31***
(***Control variables:***)				
**Sex** (male)	−.05	−.13	−.24***	−.13***
**Age** (< 25, 25–34, 35–44, 45–54, 55+)	.02	.03	−.07	.00
**Educational level** (scale from 1 ‘low’ to 4 ‘very high’)	−.04	−.03	−.05	−.06
Adjusted R square	.194	.142	.208	.204
No. cases in model	578	186	252	1009

*p ≤. 05; **p < .01; ***p < .001; no * = not significant (*p* > .05)

**Table 4:** Explaining sleep disorders – results of stepwise multiple linear regression analyses

***Dependent variable:*** Sleep disorders (single-item scale from 1 ‘not at all’ to 3 ‘severe’)	Nurses (and midwives)		Physicians		Therapists and other health professionals		All health professionals	
	*n* = 718		*n* = 222		*n* = 292		*N* = 1232	
	*beta coeff. (β)*		*beta coeff. (β)*		*beta coeff. (β)*		*beta coeff. (β)*	
	*Step 1*	*Step 2*	*Step 1*	*Step 2*	*Step 1*	*Step 2*	*Step 1*	*Step 2*
(***Independent variables:***)								
** Musculoskeletal disorders** (two-item scale from 0 ‘none’ to 4 ‘very severe’)	.24***	.11**	.12	−.01	.20***	.09	.21***	.09**
** General stress** (single-item scale from 1 ‘not at all’ to 5 ‘very strong’)	–	.33***	–	.38***	–	.44***	–	.36***
** Work stress** (ERI ratio from 0.3 ‘lowest’ to 3.7 ‘highest’)	–	.07	–	.11	–	.01	–	.06
** Physical effort at work** (three-item scale from 0 ‘lowest’ to 12 ‘highest’)	–	−.01	–	−.12	–	−.05	–	−.03
** Poor posture at work** (single-item scale from 0 ‘never’ to 4 ‘permanently’)	–	.09	–	.05	–	−.02	–	.06
(***Control variables:***)								
** Sex** (male)	.03	.05	−.04	−.05	.08	.03	.02	.01
** Age** (< 25, 25–34, 35–44, 45–54, 55+)	.14***	.15***	.11	.13	.17**	.13*	.14***	.14***
** Educational level** (scale from 1 ‘low’ to 4 ‘very high’)	.02	.02	.00	−.05	−.01	−.05	.02	−.01
Adjusted R square	.070	.211	.006	.150	.053	.217	.057	.203
No. cases in model	689	577	210	185	288	252	1189	1016

*p ≤ .05; **p < .01; ***p < .001; no * = not significant (*p* > .05)

## Discussion

In previous studies, work-related musculoskeletal and sleep disorders were both found to be relatively highly prevalent among health care and hospital workers in general and nurses in particular [2–10, 24, 25]. And both disorders were reported to be largely work-related and stress-related as well as interrelated with one another [21, 27, 30, 31]. Moreover, they were found to be a result of both psychological stress and physical strain from work. However, no studies so far have jointly investigated such disorders in association with both physical workload and psychological (work) stress, and particularly in a hospital setting in Switzerland. Against this background, this study was carried out based on self-reported survey data that were collected among health care workers and hospital employees in the German-speaking part of Switzerland.

But even though there is no other comparable observational study, particularly not for Switzerland, that has considered and included physical work factors and both general and work-related psychological stress indicators as potential risk factors for work-related MSDs and SDs, the findings of this study only partly support the results of other studies and are in part contrary to the expectations or assumptions (see research questions and Fig. 1).

In this study, as expected and often reported, severe MSDs – just like high physical workloads – are found to be significantly more common among nurses than among other health professionals. But even though the prevalence of (severe) MSDs in the present study is found to be significantly higher in nurses than in other health professions, such prevalence rates differ greatly not only between health professions but also from study to study and depending on the question or measure used or on the observation period (year or career or lifetime prevalence). A systematic review among health professionals and based on 23 retrospective and prospective observational (cross-sectional and cohort) studies [2] revealed a strong variation of 1-year prevalence rates of work-related MSDs among health professionals from 28 to 96%. Furthermore, relatively low career prevalence rates compared to 1-year prevalence rates of MSDs were reported due to recall, reporting, and/or selection bias (survivor or healthy worker effect) [2].

As assumed, MSDs are found to result from combined physical workload and psychological stress but in particular are most strongly associated with poor posture at work and general stress. This main finding is broadly consistent with two systematic reviews of 63 longitudinal (case-control or cohort) studies [14] and of 18 mainly cross-sectional studies [15]. Costa and Vieira [14] found evidence for an association or even causation between biomechanical risk factors (e.g. heavy physical work, frequent lifting, awkward posture) and/or psychosocial risk factors (e.g. low job control, high psychological work demands, high job dissatisfaction) on the one hand and different specific work-related MSDs (i.e. neck, shoulder, low back disorders) on the other. Similar to the present study findings, Long et al. [15] found that among health care workers (nurses, midwives and physicians) both psychosocial work factors or job stressors *and* physical job demands or work exposures are equally or similarly strong risk factors for work-related (upper quadrant) MSD or, more precisely, for neck, shoulder, and upper back symptoms or complaints.

Individual results of the present study are fully in line with previous findings of other studies, but some of the study findings are more surprising. Against expectations, SDs were observed to be much more a direct consequence of psychological stress particularly off the job rather than an (in-)direct result of work-related physical strain and musculoskeletal pain. Therefore, MSDs were not found to be a strong correlate or predictor of SD in a multiple adjusted and fully specified linear regression analysis. This is rather unexpected, since previous studies found clear (bivariate) associations between musculoskeletal pain and poor sleep [30, 31]. And the results also do not support previous findings and evidence from earlier studies that psychosocial work factors or job stressors such as low job control, high job strain, effort-reward imbalance, or work-life imbalance – among other things – are related to increased sleep problems [24–26]. But no other study up to now has shown what the present study has additionally found among health care workers in general and different health professionals in particular: namely, that general stress is an independent and even stronger risk factor for sleep problems than occupational stress.

Although evidence in this study is based on survey data from the workforces of just a few and not randomly selected hospitals and rehab clinics in German-speaking Switzerland and therefore strictly speaking not representative for all health professionals in Switzerland, findings of the study are nevertheless expected to be valid and generalizable. There is no indication that the study findings are substantially and systematically biased due to selection, misclassification or misinformation. And there is no plausible reason to believe that the study results would be significantly different in other parts of Switzerland or based on self-reported data from other hospitals and clinics.

Nevertheless, only about 20% of the total variance of both MSDs and SDs among health professionals were explained by psychological (work) stress and/or physical workloads. In other words, despite strong associations found and important risk factors identified there are 80% of unexplained variance remaining and therefore still some important undetected risk or explanatory factors for MSDs and SDs among health professionals. Although it was not the purpose of the present study to identify all risk factors or to explain most or as much as possible of the variance of the outcome variables, further research is obviously needed in light of such highly prevalent health problems among highly stressed and overloaded health care workers.

### Strengths and limitations

The present study has its qualities and weaknesses. One of the strengths is the heterogeneous and fairly large study population, which provided sufficient statistical power and allowed for multivariate and stratified analyses and comparisons between different health professions.

Another strength is the use of established and validated single-item or multiple-item measures for the two distinct stress concepts (general stress, work stress). All other health- and work-related measures used in this study (musculoskeletal pains, sleep disorders, physical workload) could be observed or asked directly in the survey and were easily understandable and interpretable and therefore unproblematic in view of validity. This helps to estimate and, at best, to improve the (internal and external) validity of the measures and findings. The consistent statistical approach from univariate and bivariate to multivariate analyses ensured the stability and reliability of the findings. Performing multiple adjusted, stratified, and stepwise regression analyses made it possible to test for confounding and mediation in the association analyses.

However, the cross-sectional design did not allow conclusions to be drawn concerning causation in the studied associations between exposures and outcomes. But the strong and consistent associations found across different health professions with clear dose-response relationships are at least supporting arguments when deducing causation from association.

A possible selection bias due to self-selection of the participating hospitals and rehab clinics and, in a next step, a rather low response rate among the workforces at the participating hospitals and clinics potentially calls the external validity and generalizability of the study findings into question. However, there is no indication or reason to believe that study participants or rather survey respondents differ systematically from non-respondents and, hence, that findings are systematically biased as a result of self-selection. If at all, underestimated prevalence rates (or associations) may be possible or plausible but hardly any invalid findings or completely false associations have to be expected.

## Conclusions

MSDs among health professionals in this study are found to be clearly work-related, i.e. to be primarily and quite strongly associated with physically demanding and psychologically stressful work and with general stress. In contrast, SDs have proven not to be work-related. SDs turned out to be only or mainly associated with general stress and – against expectations – only weakly associated with musculoskeletal pain and not at all with physical strain and psychological stress at work. As a result, MSDs (unlike SDs) are more prevalent among the physically burdened (hospital) nurses than among other health care workers. Preventing work-related MSDs or rather combined and accumulated (low) back, neck and shoulder pain therefore requires mainly a reduction of the physical workload, whereas SDs or more precisely severe problems in falling or staying asleep can be prevented most effectively by reducing the general stress level.

## Data Availability

Secondary data used in this study were collected anonymously and on a voluntary basis and by random and full sample surveys among the workforces of four public hospitals and two privately operated rehab clinics. Nevertheless, the hospitals and clinics have agreed to participate in the survey on condition that the collected data may not passed on to any third parties. Or more precisely, data are not publicly accessible and freely available since the use and analysis of the pooled data and the publication of any research findings and study results out of it are restricted by contract with the participating companies (hospitals, clinics) to the University of Zurich (Epidemiology, Biostatistics and Prevention Institute) and the collaborating Careum Research, a division of the Careum Foundation. The use of the data therefore is strictly limited to the responsible scientists from the two research institutions.

## Abbreviations

MSDs: Musculoskeletal disorders
SDs: Sleep disorders
ERI: Effort-reward imbalance
